# Pro-vaccination Groups Expressing Hesitant Attitudes: A Cross-Sectional Study About the Difference Between Attitudes and Actual Behavior in Israel

**DOI:** 10.3389/fpubh.2022.871015

**Published:** 2022-04-27

**Authors:** Rana Hijazi, Anat Gesser-Edelsburg, Paula Feder-Bubis, Gustavo S. Mesch

**Affiliations:** ^1^School of Public Health, University of Haifa, Mount Carmel, Haifa, Israel; ^2^Head of the Health Promotion Program and Head of the Health and Risk Communication Lab, School of Public Health, University of Haifa, Mount Carmel, Haifa, Israel; ^3^Department of Health Policy and Management, Faculty of Health Sciences and Guilford Glazer Faculty of Business and Management, Ben-Gurion University of the Negev, Be'er Sheva, Israel; ^4^Department of Sociology, University of Haifa, Mount Carmel, Haifa, Israel

**Keywords:** pro-vaccination groups, hesitant attitudes, actual behavior, parents, children (3–5 years), Israel, cross-sectional study

## Abstract

**Background:**

Vaccines have contributed to the decline in mortality, morbidity, and even the eradication of various infectious diseases. Over time, the availability of information to the public and the request for public involvement in the health decision-making process have risen, and the confidence in vaccines has dropped. An increasing number of parents and individuals are choosing to delay or refuse vaccines.

**Objectives:**

(1) Identifying hesitant attitudes among pro-vaccination parents; (2) testing the difference between the rate of hesitant attitudes and the rate of hesitancy in practice among pro-vaccination parents; and (3) examining the association of sociodemographic characteristics (gender, age, marital status education and religious affiliation) with the difference between hesitant attitudes and hesitancy in practice among pro-vaccination parents.

**Methods:**

Descriptive cross-sectional survey using an online survey that measured vaccine hesitancy among pro-vaccination parents (*n* = 558) whose children were in kindergarten (3–5 years), according to a variety of sociodemographic characteristics.

**Results:**

A significant difference was found between the rate of hesitant attitudes and the rate of hesitation in actual vaccination among pro-vaccination and hesitant parents, where despite that 26% of the parents had hesitant attitudes, only 19% hesitated in practice [*P* = 0.0003]. There was also a significant difference between the rate of hesitant attitudes and the rate of hesitancy in practice among women [*P* = 0.0056] and men [*P* = 0.0158], parents between 30 and 39 years of age [*P* = 0.0008], traditional parents [*P* = 0.0093], Non-academic parents [*P* = 0.0007] and parents with BA degree [*P* = 0.0474].

**Conclusion:**

Pro-vaccination individuals may have hesitant attitudes regarding vaccines. Therefore, it is very important for health authorities to address the public's fears and concerns, including those who are classified as pro-vaccination.

## Introduction

Vaccines are considered one of the most important achievements of public health in the 20th century. Vaccination programs have contributed to the decline in mortality, morbidity and have even aided in the eradication of various infectious diseases (1). The success of vaccination programs relies on high vaccination coverage, which leads to direct protection for vaccinated individuals and indirect protection to the overall community. Over time, the confidence in vaccines has dropped. The availability of information drives the parents to act as informed consumers and motivates them to research vaccines to make informed decisions. Parents who “do the research” and make an individualized and informed decision are described as “good parents” (2). As a result, an increasing number of parents and individuals are choosing to delay or refuse vaccines, causing resurgent outbreaks of vaccine-preventable diseases like measles and diphtheria, and drawing attention to vaccine-hesitant groups (3–6). In 2019, the World Health Organization included vaccine hesitancy as one of the ten threats to global health of the year (7).

There is a lack of consistency regarding the definition of “Vaccine Hesitancy” (8, 9). The Strategic Advisory Group of Experts (SAGE) on Immunization of the World Health Organization (WHO) defined vaccine hesitancy as a behaviorally-related definition which “refers to delay in acceptance or refusal of vaccines despite availability of vaccination services” (5, 9, 10). Researchers like Bedford, Benin and Brewer argue that SAGE's definition of vaccine hesitancy as behavior is lacking since it does not take into consideration those who vaccinate according to the recommended schedule, yet still have concerns about doing so (8, 11–15). These researchers asked for a more precise definition of the term “vaccine hesitancy”, and suggested that the hesitancy is not only behavioral but also psychological. Therefore, it is difficult to depict a clear picture of vaccine hesitancy at the population level because hesitancy is not related directly to vaccine uptake and vaccine-hesitant individuals may accept all recommended vaccines (16). Vaccine-hesitant individuals are a heterogenous group within this continuum (7, 17).

Recent studies focus on the “3Cs” model to describe factors influencing vaccine hesitancy decision-making process beyond the sociodemographic characteristics. This model suggests that complacency, convenience, and confidence are factors that influence vaccine hesitancy (9, 10). The “3Cs” model was adopted by SAGE as the most useful model to assess the determinants of vaccine hesitancy. Complacency exists where perceived risks of vaccine-preventable diseases are low and vaccination is not considered a necessary preventive action. It is influenced by many factors including other life or health responsibilities that may be more of a priority at that point in time. Confidence is defined as a trust in the effectiveness and safety of vaccines, trust in the system that delivers them, and the motivations of the policymakers who decide on the needed vaccine. Vaccine convenience is measured by the extent to which physical availability, affordability and willingness-to-pay, geographical accessibility, ability to understand (language and health literacy) and appeal of immunization services affect uptake (10). Despite this, some studies perceive that the “3Cs” model is too narrow and should be expanded (18).

Previous studies indicated that sociodemographic characteristics influence vaccine hesitancy. Issues around religion, culture, gender, socioeconomic status, marital status (19) and the number of children (20) were common. Authors have shown a significant disagreement about the role of socioeconomic status and education on vaccine hesitancy. Analysis of vaccine hesitancy according to income status indicated that in low- and lower-middle-income countries, a lack of services was the main contributor to vaccine hesitancy. Yet recent data indicates that vaccine hesitancy affects all countries regardless of income status (21). Even in countries with publicly funded national vaccination programs, vaccine hesitancy occurs as a result of socioeconomic inequalities (22). Socioeconomic status was found in several studies as a determinant of hesitancy. These studies suggest that vaccine hesitancy occurs across those with low socioeconomic status (23, 24) and economic hardship (25). Studies that investigated the effect of parents' education on vaccine hesitancy presented a mixed set of results (21). For example, Yaqub et al. (24) and Larson et al. (23) suggest that vaccine hesitancy occurs amongst university-educated middle-class people. While Bertoncello et al. (25) suggest that there is no association between parents' education and vaccine hesitancy, and a higher level of education seems to be a protective factor against refusing vaccines.

Moreover, several studies examining various populations and different vaccines have indicated that vaccine hesitancy is related to, but not limited to, prior beliefs about vaccinations (26, 27), perceived benefits of vaccines (28), attitudes toward vaccines (29, 30), whether the child has been previously vaccinated (29) and previous experiences with vaccinations (31). Social norms play an important role in vaccination-related decisions (32). Vaccination as a social norm was found to be a potentially powerful driver of vaccine acceptance in studies over the years (16, 33). These studies have repetitively highlighted the importance of the perceptions of social norms (i.e., belief that others vaccinate) in vaccination promotion (32, 34), and suggested that parents vaccinate their children because vaccination is considered a social norm (35). Moreover, the perception of social consensus and unity of norms regarding vaccination increases the tendency of individuals to adhere to the perceived social norm (36–38) and conform to authorities and health professionals (4).

Given the growth of the vaccine hesitancy phenomenon and its critical consequences for public health, scholars have devoted significant attention to understand vaccine hesitancy in recent years (39). Despite the wide range of findings, the results of several studies, as mentioned above, and several systematic reviews suggest that there are still factors contributing to vaccine hesitancy to be identified and further explored (4, 40, 41). Moreover, as we mentioned above there are some discrepancies among scholars in terms of what exactly falls under the umbrella of “vaccine hesitancy” (42). In light of the debate concerning the definition of vaccine hesitancy and the fact that there are few empirical studies focusing on individuals who vaccinate according to the recommended schedule but still have concerns, the study aims to examine hesitant attitudes among pro-vaccination parents.

In Israel, vaccine coverage is widespread. The reported vaccination rates are high and meet the WHO's goals (43). However, recent studies point to a growing concern regarding the speared of vaccine hesitancy among parents in Israel. Vaccine hesitancy become a more prevalent phenomenon in recent years and is considered a public health concern in Israel (43–46). Previous Israeli studies found that vaccine hesitancy is associated with higher education, affirming that vaccine hesitancy is an informed decision and not caused by objective barriers of cost and availability (47, 48). In addition, a recent study indicates that vaccination acceptance among the Israeli population relies on risk-benefit perception which may be influenced by misinformation regarding vaccines (49).

This study objectives are: (1) Identifying hesitant attitudes among pro-vaccination parents; (2) testing the difference between the rate of hesitant attitudes and the rate of hesitancy in practice among pro-vaccination parents; and (3) examining the association of sociodemographic characteristics (gender, age, marital status education and religious affiliation) with the difference between hesitant attitudes and hesitancy in practice among pro-vaccination parents.

## Methods

### Research Design and Procedure

A descriptive cross-sectional study using an online survey that measured vaccine hesitancy among pro-vaccination parents according to a variety of sociodemographic characteristics, following the STROBE guidelines (50) for cross-sectional studies (see Supplementary Material 1).

In the first stage, we developed a questionnaire and pilot tested it with 20 pro-vaccination parents for their comprehension and interpretation of survey questions and to enable the examination and description of why hesitancy occurs so that we could test the hesitancy scale in the survey. Pro-vaccination parents were defined according to their answer to a filtering question. The participants were asked if they give their children all the vaccines, according to the routine vaccination schedule. Participants who vaccinated their children with all vaccines according to the routine vaccination schedule were considered “pro-vaccination”.

The study was approved by the Faculty of Social Welfare and Health Sciences Ethics Committee for research with human subjects at the University of Haifa (approval no. 421/17).

### Sampling and Data Collection

An online survey was distributed in January 2020 to a panel of samples that represented the adult population in Israel. The participants were sampled from iPanel, an Israeli internet panel. The online survey was designed using the Qualtrics XM online survey (Qualtrics Survey Software) that enabled the rapid and effective distribution of an online questionnaire to our research population.

In the first stage, we examined the size and proportion of each of the three groups (pro-vaccination, hesitant, and anti-vaccination) in the adult population in Israel. As a representative sample of this population (*N* = 500), they were asked if they give their children all of the vaccines according to the routine vaccination schedule. If they answered “yes”, the participant was considered a pro-vaccination parent. If they answered “no”, the participant was considered an anti-vaccination parent. However, in order to consider the participants vaccine-hesitant, they needed to answer: “I'm selective in vaccinating my children” or “I give my children all of the vaccines, but not according to the routine vaccination schedule”.

After learning about the size of the three groups, we decided to exclude the anti-vaccination group of participants because it represented a very small minority. The sample size of the entire population from which the groups were sampled for the comprehensive study was determined, so that the differences between the groups–if found–would be significant. Accordingly, we decided to approach 600 participants from a representative sample of the general population (from which we excluded the anti-vaccination group and those who refused to answer the questionnaire).

We tried to approach each sampled individual up to three times in the sampling process. If the sampled individual did not respond to the questionnaire after three attempts, we sent the questionnaire to another individual from the same age group. The response rate was 37%. A total of 570 parents whose children were in kindergarten (3–5 years) were filtered out of the representative samples of the population in Israel and classified by their vaccination status. The rationale behind selecting parents whose children are in kindergarten stems from the relevance of vaccines to this specific population. By recruiting the aforementioned parents, we were also able to avoid recall bias.

In order to determine the participants' vaccination status, they were asked if they give their children all of the vaccines according to the vaccination routine. If they answered “yes,” the participant was considered a pro-vaccination parent. If they answered “no,” the participant was considered an anti-vaccination parent. However, to consider the participants as vaccine-hesitant, they needed to answer: “I'm selective in vaccinating my children” or “I'm vaccinating my children all of the vaccines, but not according to the vaccination routine schedule”.

451 participants were pro-vaccination, 107 were hesitant, and 12 participants were anti-vaccination. Other studies also list these groups in a similar order. However, in recent years, vaccine-hesitant groups are growing. Pro-vaccination groups are still the largest group in the population, and anti-vaccination groups are still the smallest group (51, 52).

The anti-vaccination participants were excluded from the study because they were a statistically small group, insignificant to the study, and not the target audience. As a result, only the remaining 558 participants were included in the study.

### Research Tools

A scale of vaccine hesitancy was designed to identify attitudes regarding vaccines' effectiveness and importance to examine the relationship between hesitant attitudes and actual vaccination behavior. The scale was based on a previously validated scale of vaccine hesitancy (23).

### Questionnaire Structure and Variable Design

All of the sociodemographic characteristics (gender, marital status, education, and religious affiliation) were each marked by the study participants out of Pre-defined sub-categories list that were presented in the questionnaire for each variable, except for age, which was filled out by the study participants.

In the first part of the questionnaire, there was a filtering question that asked if the participant was a parent of a child in kindergarten (3–5 years old). If the participants met the inclusion criteria, they were moved to the second part of the questionnaire and asked to fill out their demographic information and asked if they give their children all the vaccines according to the nationally stipulated vaccination schedule. The third part of the questionnaire was based on a validated scale on vaccine hesitancy (23) and included an index of seven 5-point Likert scale statements in which the participants were requested to indicate how much they agree or disagree with each statement (Cronbach α = 0.91). The statements focused on the effectiveness and importance of routine vaccines (Table 1). The score of the 5-point Likert scale indicates the level of hesitation. An index score of 2 and below indicates low hesitation in regard to vaccines. However, a high hesitation score should be above 2.

**Table 1:** Vaccine hesitancy 5-point Likert Scale questions.

**How much do you agree with each of the following statements? Please indicate your response with a check mark (√) in the appropriate box, using the scale**	**1 (strongly disagree)**	**2 (disagree)**	**3 (neither agree nor disagree)**	**4 (agree)**	**5 (strongly agree)**
1. Routine childhood vaccines are important for my child's health.					
2. Childhood routine vaccines are effective.					
3. Having my child vaccinated is important for the health of others in my community.					
4. All childhood routine vaccines offered by the health ministry are beneficial.					
5. The information I receive about vaccines from vaccine programs are reliable and trustworthy.					
6. Getting vaccinated is a good way to protect my child/children from diseases.					
7. Generally, I do what my doctor or health ministry recommends regarding vaccines for my child/children.					

### Credibility and Validity

In order to quantitively measure and address vaccine hesitancy attitudes, we conducted a pilot study. We used questions from a validated survey tool on vaccine hesitancy (23) and culturally adapted the questions to the Israeli population. The questions were written in Hebrew and translated to Arabic, so the participants could choose the language of the questionnaire. The pilot study included 20 participants who were asked to fill out the questionnaire and provide feedback on the wording of the questions and the time needed to answer the questionnaire. Changes were made accordingly after the pilot study, before distributing the questionnaire.

### Analysis

The hesitancy index was grouped into two levels to examine the relationship between hesitant attitudes and actual vaccination:

- Low hesitation (index score 2 and below).
- High hesitation (index score above 2).

The Chi-square test for independence was used to test the difference between the sociodemographic characteristics (gender, age, marital status, education, and religious affiliation) in relation to the rate of hesitant attitudes.

In order to test the difference between the rate of hesitant attitudes and the rate of hesitancy in practice, we used McNemar's statistic test for dependent proportions. We conducted the test on the whole sample in order to identify the effect of their individual characteristics on their attitude toward vaccination. We analyzed the data according to the participants' sociodemographic characteristics.

## Results

The study participant included 125 (22.4%) males and 433 (77.6%) females. Most of the study participants (57.3%) were between 30 and 39 years of age, 22.6% were between 18 and 29 years of age, and 19.4% were between 40 and 49. The majority (79.9%) of the study participants were Jewish and 17.2% were Arab. The majority (90.5%) of the study participants are married, and 58.9% with a BA (41.0%) or MA (17.9%) degree. As per religious affiliation, 252 (21.7%) are seculars, 121 (21.7%) traditional, 108 (19.4%) religious, and 76 (13.6%) Ultra-Orthodox Jews (Table 2).

**Table 2:** Sociodemographic characteristics of the survey participants (*n* = 558).

**Characteristic**	**Sub characteristic**	**Frequency**	**Percentage (%)**
Gender	Male	125	22.4
	Female	433	77.6
Age (years)	18–29	126	22.6
	30–39	320	57.3
	40–49	108	19.4
	≥50	4	0.7
Ethnicity	Jewish	446	79.9
	Arab	96	17.2
	Druze	13	2.3
	Other	3	0.5
Marital status	Single	12	2.2
	Married	505	90.5
	Divorced	16	2.9
	Unmarried single parent	4	0.7
	Parent in a relationship	21	3.8
Education	Primary school	9	1.6
	Secondary	73	13.1
	Post-secondary	138	24.7
	BA	229	41.0
	MA	100	17.9
	Ph.D.	5	0.9
	Other	4	0.7
Religious affiliation	Secular	252	45.2
	Traditional	121	21.7
	Religious	108	19.4
	Ultra-orthodox Jew	76	13.6
	Other	1	0.2

First, the Chi-square test for independence was used to test the difference between sociodemographic characteristics and the rate of hesitant attitudes. All the sociodemographic characteristics were found nonsignificant for gender [χ^2^(1) = 2.84, *P* = 0.0920], age [χ^2^(2) = 3.61, *P* = 0.1646], religious affiliation [χ^2^(3) = 3.19, *P* = 0.3637], and education [χ^2^(2) = 1.08, *P* = 0.5837].

The study included 558 participants, 451 participants were pro-vaccination and 107 were hesitant (see Table 3). The McNemar's test for dependent proportions was used to test the difference between the rate of hesitant attitudes and the rate of hesitancy in practice within the total sample. A significant difference was found between the rate of hesitant attitudes and the rate of hesitation in actual vaccination, where 26% of the parents have hesitant attitudes, but only 19% hesitate in practice [χ2(1) = 13.0, *P* = 0.0003].

**Table 3:** Hesitant attitudes toward vaccination vs. vaccination in practice (using McNemar's test).

**Hesitancy Index**	**Pro (Fully vaccinated)^a^**		**Hesitant (Partially vaccinated)^a^**		**Total**		**McNemar's test Statistic**
**Pro (Index score** ** < = ** **2)**	373	66.85%	39	6.99%	412	73.84%	^χ2^(1) = 13.0 *P* = 0.0003
**Hesitant (Index score** **>** **2)**	78	13.98%	68	12.19%	146	26.16%	
**Total**	451	80.82%	107	19.18%	558	100.0%	

a *Percentages in the above table are within the total sample (N = 558)*.

In the third stage, McNemar's test for dependent proportions was used to test the difference between the rate of hesitant attitudes and the rate of hesitancy in practice by background characteristics (Table 4). A significant difference was found among women, whose rate of hesitant attitudes is 24.5% and the rate of hesitancy in practice is only 18.5% [χ^2^(1) = 7.68, *P* = 0.0056]. A significant difference was also found among men, whose rate of hesitant attitudes is 32.0% and their rate of hesitancy in practice is 21.6% [χ^2^(1) = 5.83, *P* = 0.0158]. A significant difference was found parents between 30 and 39 years of age, where the rate of hesitant attitudes is 25.0% and the rate of hesitancy in practice is 16.9% [χ^2^(1) = 11.27, *P* = 0.0008]. Within the religious demographic, a significant difference was found among traditional participants, where the rate of hesitant attitudes is 24.8% and the rate of hesitancy in practice is only 14.1% [χ^2^(1) = 6.76, *P* = 0.0093]. However, the difference was nonsignificant among secular, religious, and ultra-orthodox participants. When education was tested, we found a significant difference between the rate of hesitant attitudes and the rate of hesitancy in practice among Non-academic participants [χ^2^(1) = 11.36, *P* = 0.0007] and among participants with a BA degree [χ^2^(1) = 3.93, *P* = 0.0474]. The rates of hesitancy were 28.2% and 25.8% and the rates of hesitancy in practice were 16.8% and 20.1% among Non-academic participants and participants with bachelor's degrees, respectively.

**Table 4:** Hesitant attitudes toward vaccination vs. vaccination in practice by sociodemographic characteristics (using McNemar's test).

**Sociodemographic characteristic**		**Hesitancy attitude**	**Pro (Fully vaccinate)**		**Hesitant (Partially vaccinate)**		**Total**		**McNemar's test Statistic**
**Gender**	**Female**	Pro (Hesitancy Index < = 2)	296	68.4%	31	7.2%	327	75.5%	χ^2^(1) = 7.68 *P* = 0.0056
		Hesitant (Hesitancy Index > 2)	57	13.2%	49	11.3%	106	24.5%	
		Total	353	81.5%	80	18.5%	433	100.0%	
	**Male**	PRO (Hesitancy Index < = 2)	77	61.6%	8	6.4%	85	68.0%	χ^2^(1) = 5.83 *P* = 0.0158
		Hesitant (HesitancyIndex > 2)	21	16.8%	19	15.2%	40	32.0%	
		Total	98	78.4%	27	21.6%	125	100.0%	
**Age (years)**	**18–29**	PRO (Hesitancy Index < = 2)	89	70.6%	8	6.4%	97	77.0%	χ^2^(1) = 3.85 *P* = 0.0500
		Hesitant (Hesitancy Index > 2)	18	14.3%	11	8.7%	29	23.0%	
		Total	107	84.9%	19	15.1%	126	100.0%	
	**30–39**	PRO (Hesitancy Index < = 2)	223	69.7%	17	5.3%	240	75.0%	χ^2^(1) = 11.27 *P* = 0.0008
		Hesitant (Hesitancy Index > 2)	43	13.4%	37	11.6%	80	25.0%	
		Total	266	83.1%	54	16.9%	320	100.0%	
	**40+**	PRO (Hesitancy Index < = 2)	61	54.5%	14	12.5%	75	67.0%	χ^2^(1) = 0.29 *P* = 0.5900
		Hesitant (Hesitancy Index > 2)	17	15.2%	20	17.9%	37	33.0%	
		Total	78	69.6%	34	30.4%	112	100.0%	
**Religious Affiliation**	**Secular**	PRO (Hesitancy Index < = 2)	169	67.1%	18	7.1%	187	74.2%	χ^2^(1) = 3.45 *P* = 0.0633
		Hesitant (Hesitancy Index > 2)	31	12.3%	34	13.5%	65	25.8%	
		Total	200	79.4%	52	20.6%	252	100.0%	
	**Traditional**	PRO (Hesitancy Index < = 2)	85	70.3%	6	5.0%	91	75.2%	χ^2^(1) = 6.76 *P* = 0.0093
		Hesitant (Hesitancy Index > 2)	19	15.7%	11	9.1%	30	24.8%	
		Total	104	86.0%	17	14.1%	121	100.0%	
	**Religious**	PRO (Hesitancy Index < = 2)	74	68.5%	9	8.3%	83	76.9%	χ^2^(1) = 0.73 Exact *P* = 0.5235
		Hesitant (Hesitancy Index > 2)	13	12.0%	12	11.1%	25	23.2%	
		Total	87	80.6%	21	19.4%	108	100.0%	
	**Ultra-orthodox**	PRO (Hesitancy Index < = 2)	44	57.9%	6	7.9%	50	65.8%	χ^2^(1) = 3.86 Exact *P* = 0.0784
		Hesitant (Hesitancy Index > 2)	15	19.7%	11	14.5%	26	34.2%	
		Total	59	77.6%	17	22.4%	76	100.0%	
**Education**	**Non-academic**	PRO (Hesitancy Index < = 2)	143	65.0%	15	6.8%	158	71.8%	χ^2^(1) = 11.36 *P* = 0.0007
		Hesitant (Hesitancy Index > 2)	40	18.2%	22	10.0%	62	28.2%	
		Total	183	83.2%	37	16.8%	220	100.0%	
	**BA**	PRO (Hesitancy Index < = 2)	155	67.7%	15	6.6%	170	74.2%	χ^2^(1) = 3.93 *P* = 0.0474
		Hesitant (Hesitancy Index > 2)	28	12.2%	31	13.5%	59	25.8%	
		Total	183	79.9%	46	20.1%	229	100.0%	
	**MA/PhD**	PRO (Hesitancy Index < = 2)	72	68.6%	9	8.6%	81	77.1%	χ^2^(1) = 0.00 Exact *P* = 1.0000
		Hesitant (Hesitancy Index > 2)	9	8.6%	15	14.3%	24	22.9%	
		Total	81	77.1%	24	22.9%	105	100.0%	

## Discussion

The professional literature is divided on the definition of what is hesitation in vaccines. Some definitions of vaccine hesitancy are solely based on the actual behavior of individuals and the barriers that prevent them from getting vaccinated or vaccinating their children regardless of their attitudes (5, 9, 10, 17), while other definitions take into account hesitant attitudes (53).

However, most theories and empirical studies divided groups according to their vaccination behavior, which is pro-vaccination, hesitant and anti-vaccination, regardless of their attitudes about vaccines. Some studies indicate that vaccine-hesitant groups have been growing in recent years However, pro-vaccination groups are still the largest group in the population, and anti-vaccination groups are still the smallest group (51, 52).

Few studies focused on pro-vaccination groups with hesitant attitudes about vaccines (54, 55). This study sought to contribute to the literature by revealing the gap between hesitant attitudes and actual behavior in pro-vaccination groups.

Different interpretations can be offered to explain the gap between hesitant attitudes and behavior. We would like to discuss two of them: social norms, and risk perception. One of the main components in the literature that predicts behavior is social norms. Since there is a very high percentage of childhood vaccines uptake in Israel (more than 90%), it is possible that parents who feel worries and concerns are persuaded to do so because the vaccines are accepted in society. Conformity theory can contribute to understanding the importance of the norm component in responding to vaccines. According to this theory, social groups often penalize individuals who deviate from accepted norms. As a result, individuals tend to suppress their individuality and conform to social norms to maintain their social acceptance and popularity (56). Therefore, most parents would like to vaccinate via comparing their payoffs with others' (57). The behavior, opinions, recommendations, and advice given by significant others such as friends, family members, and trusted colleagues can affect the parents' decision to vaccinate (58–60). Moreover, public policies (60) and health professionals tacitly motivate parents to vaccinate and conform to social norms (61). In other words, despite that vaccination may be voluntary in theory, health professionals consider vaccination compulsory. Health professionals give parents information that is not only designed to advise but also induce conformity, including not all the information about possible risks or side effects, about the duration of protection or systemic effects on their child's immune system (61, 62). In addition, the ongoing debate regarding required immunization as a condition for children's admission into the educational system puts some parents under social pressure (63). This debate makes it difficult for parents to ask questions regarding vaccinations and to come to a reasonable decision on their own (61).

The second possible explanation for the gap between attitudes and behavior may lie in risk perception. It may be that parents choose to vaccinate their children despite their concerns because of their low-risk perception of the adverse effects of vaccines and high-risk perception of disease infection. Low-risk perception of adverse effects is due to the low incidence of serious adverse effects after vaccination. Additionally, a lack of accounts regarding any injuries in children in their narrow and wide circles contributes to their low-risk perception. Some studies support this finding. These studies suggest that determinants such as the perceived risk of disease infection (24, 58, 64), the perceived safety and efficacy of vaccine (e.g., vaccine side-effects and the related adverse complications) (65, 66), as well as the social and financial costs associated with vaccination and disease infection (e.g., the charge of vaccine administration, expenses for infection treatment, and absence from work) (67) all contribute to an individual's decision-making process regarding vaccines.

On one hand, vaccinating their children could lead to potential side effects. On the other hand, not vaccinating could put their children at risk of getting infected from vaccine-preventable diseases, risk rejection from some schools, and disrupt herd immunity by affecting other children. This may be explained by a phenomenon called “optimism bias” (68–70). This phenomenon suggests that when it comes to predicting what will happen to us in the future, we overestimate the likelihood of positive events and underestimate the likelihood of negative events (71). In the context of vaccines, parents tend to be more optimistic regarding vaccinating their children and underestimate the likelihood of the vaccine's serious adverse effects.

Sociodemographic characteristics influenced the difference between hesitant attitudes and vaccination uptake. Most published studies evaluating parental vaccine hesitancy focused on one specific vaccine (like influenza, HPV, or MMR vaccines) and the effect of socioeconomic and educational variables on vaccination decision-making (19, 42, 72). The study aimed to examine the effect of additional sociodemographic characteristics on the rate of vaccine hesitancy in general and not limit our study to a specific vaccine.

In this study, both mothers and fathers were found to be more hesitant in their attitude than in their behavior. Therefore, gender was found to be not an influencing factor to the difference between hesitant attitudes and vaccination uptake.

Besides, again this study was found to be influencing the difference between hesitant attitudes and vaccination uptake. There was a significant difference in the rate of hesitant attitudes and the rate of hesitancy in practice among parents between the ages of 30–39. The explanation for this is that not only do most young women and men become parents in this age range, but they also have more accessible attitudes regarding vaccines, which allows them to think about vaccines much more frequently than other age groups (73).

Moreover, there was no significant difference in the rate of hesitant attitudes and the rate of hesitancy in practice among older parents ages 40 and above. This finding may be attributed to the experience accumulated over time on the importance of vaccination. Some parents in these age groups generally have more than one child. Therefore, they may have had a positive experience vaccinating their children without serious adverse effects. As a result, older parents have higher confidence in vaccine safety and efficacy (74). Consistent with this finding, Larson and colleagues found that individuals between the age of 18–24 years are more likely to believe that vaccines are safe compared to 25–34-year-olds and that individuals over 65 years old are more likely to report that vaccines are effective (75).

Regarding religion, a significant difference was found between hesitant attitudes and vaccination uptake among traditional parents. The classification of religiosity among the Jewish and Arab populations is different. The Arab population is divided into not religious, not so religious, religious, and very religious (76). While Jewish population is divided into different set groups: secular, traditional, religious, very religious, and ultra-Orthodox. The traditional Jewish group is situated on a spectrum somewhere between religious and secular. For the most part, traditional Jews observe specific commandments and traditions considered to be clear signs of traditional belief. They do not necessarily comply strictly with Jewish law but rather out of a sense of identification and belonging with the Jewish people or out of a belief that these traditional values must be safeguarded to guarantee the existence of the Jewish people (77). Their characteristics and lifestyle are different than religious and ultra-orthodox groups. Traditional people go online and use smartphones like secular people. Therefore, they are apparently more exposed to the debate of questionable information regarding vaccine safety on social media as opposed to, Ultra-orthodox.

In line with previous studies, this study found that the level of education is a contributing factor to vaccine hesitancy. There was a higher consistency between hesitant attitudes and vaccination uptake among parents with graduate degrees. The higher the parents' education level, the smaller the difference between hesitant attitudes and vaccination uptake. A significant difference in the rate of hesitant attitudes and the rate of hesitancy in practice was found among Non-academic parents and parents who hold a bachelor's degree. Studies by Opel et al. and Smith et al.'s show a similar finding. More concerns and refusal of childhood vaccines were found among college-educated couples and parents with lower education levels (19, 78). For example, parents who held a master's degree or a doctoral degree had less positive views on vaccine importance and effectiveness (75), which may be due to the fact that these parents have a higher level of health literacy. Therefore, highly educated parents tend to make reasoned and informed decisions based on the literature they choose to read (61).

### Study Limitations

Participant recruitment may be a limitation in this study. Despite that the sample of the study is representative, it only included participants who participated by choice, and this is an indicator of selection bias. Given the growth of the vaccine hesitancy phenomenon, further studies should be conducted to examine the gap between vaccination behavior in different populations and groups with different attitudes regarding vaccination. In addition, exploring how additional socio-demographic characteristics affect vaccination behavior and attitudes in different populations is crucial to understanding the phenomenon of vaccine hesitancy.

## Conclusion

Pro-vaccination individuals may have hesitant attitudes regarding vaccines. However, they eventually choose to get vaccinated or vaccinate their children probably because of positive experiences with vaccines in the past, social norms, risk perception, and responsibility. However, some people from this group may express concerns and worries regarding vaccines. They may report, as they did in this study, that the information they receive about vaccines from vaccine programs (authorities and health experts) is not fully reliable and trustworthy. They also may experience pressure from authorities and their social circle to get vaccinated or to vaccinate their children. This phenomenon can turn into a boomerang effect because as we have found in another study, people who express hesitant attitudes can eventually become hesitant in behavior (52).

We recommend that health authorities and policymakers would address the fears and concerns of all groups, including those who are pro-vaccination, taking into consideration the difference between hesitant attitudes and hesitancy in practice among pro-vaccination parents, based on sociodemographic characteristics (gender, age, marital status education, and religious affiliation).

## Data Availability Statement

The raw data supporting the conclusions of this article will be made available by the authors, without undue reservation.

## Ethics Statement

The studies involving human participants were reviewed and approved by the Faculty of Social Welfare and Health Sciences Ethics Committee for research with human subjects at the University of Haifa (approval no. 421/17). The patients/participants provided their written informed consent to participate in this study.

## Author Contributions

RH carried out this research as part of her PhD dissertation under the supervision of AG-E and GSM. RH conceptualized the study, reviewed the literature, conducted the data analysis, written the manuscript, and took full responsibility for the study. AG-E provided input on the study conceptualization, data analysis, and writing the first drafts of the manuscript. GSM and PF-B critically reviewed the manuscript and helped shape the final version of the manuscript. All authors approved the final manuscript.

## Conflict of Interest

The authors declare that the research was conducted in the absence of any commercial or financial relationships that could be construed as a potential conflict of interest.

## Publisher's Note

All claims expressed in this article are solely those of the authors and do not necessarily represent those of their affiliated organizations, or those of the publisher, the editors and the reviewers. Any product that may be evaluated in this article, or claim that may be made by its manufacturer, is not guaranteed or endorsed by the publisher.

## Supplementary Material

Supplementary Material 1 STROBE statement.
